# Supplementary material for: “Chasing the pain relief, not the high”: Experiences managing pain after opioid reductions among patients with HIV and a history of substance use
Source: PLoS One. 2020 Mar 25;15(3):e0230408. doi: 10.1371/journal.pone.0230408 (PMC7094831; doi:10.1371/journal.pone.0230408)
Supplement: Attachment — Submitted filename: Response to Reviewers.docx [file pone.0230408.s001.docx]

| **PONE-D-19-16726** | | |
| --- | --- | --- |
| **“Chasing the pain relief, not the high”: experiences managing pain after opioid reductions among patients with HIV and a history of substance use** | | |
| **PLOS ONE** | | |
|  |  |  |
| **1. Is the manuscript technically sound, and do the data support the conclusions?** | | |
| Reviewer 1: Yes |  |  |
| Reviewer 2: Yes |  |  |
| **2. Has the statistical analysis been performed appropriately and rigorously?** | | |
| Reviewer 1: Yes |  |  |
| Reviewer 2: Yes |  |  |
| **3. Have the authors made all data underlying the findings in their manuscript fully available?** | | |
| Reviewer 1: Yes |  |  |
| Reviewer 2: No |  |  |
| **4. Is the manuscript presented in an intelligible fashion and written in standard English?** | | |
| Reviewer 1: Yes |  |  |
| Reviewer 2: Yes |  |  |
|  |  |  |
| **COMMENTS** | **RESPONSE** | **LOCATION OF REVISION IN REVISED MANUSCRIPT WITH TRACK CHANGES** |
| **REVIEWER 1** |  |  |
| ***Overall*** |  |  |
| This is a qualitative analysis of interviews with individuals with HIV who experienced after long term opioid pain medication reduction or discontinuation. This is an important contribution to the field and is generally a well-done analysis with an accompanying well-written manuscript. | N/A | N/A |
| ***Abstract*** |  |  |
| Consider reporting Ns rather than percentages in the results paragraph (see related comment regarding Results below). | All percentages in the abstract have been changes to Ns. | Abstract, pg 1 |
| ***Background*** |  |  |
| The final paragraph in the introduction would benefit from introducing the participant population as having a history of illicit substance use in addition to HIV and chronic pain, as this is an important differentiating factor for the sample. | We changed the second to last sentence in the final paragraph of the introduction to state "The aim of this study is to qualitatively explored chronic pain management experiences among PLWH **with a history of illicit substance use** after long-term opioid therapy reductions or discontinuations to better understand. We focus specifically on the patients’ rationales for and descriptions of barriers and facilitators to pain management modalities." | Pg 3, line 60-63 |
| ***Methods*** |  |  |
| Please provide a citation for the longitudinal COPING study cohort, if one exists | COPING data has not yet been published so there is no citation to include at this time | N/A |
| Please provide additional information about how OPR dose reduction or discontinuation was defined? Participant self report vs chart review? | We included the phrase "as determined by a medical chart review" to the end of the final sentence of the paragraph titled, "Study Sample" to help clarify how we determined dose reduction/discontinuation. | Pg 4, lines 75-76 |
| Please provide some additional information about how/when interim analyses were conducted, which would allow the investigators to know when thematic saturation was reached. | Interim analyses were conducted on an on-going basis throuhgout the entire interview process. We changed the first sentence of the Data Collection and Analysis section to reflect this: "We qualitatively analyzed conducted 18 semi-structured interviews with ongoing interim analyses throughout the interview process, stopping once theoretical saturation was reached." | Pg 4, lines 79-81 |
| Please provide citations for content analysis methods (line 79). | We've added two citations for content analysis into the methods section | Pg 4, line 81 |
| ***Results*** |  |  |
| Given that this is a qualitative analysis with an appropriately small N, I would recommend reporting the Ns for each demographic characteristic (ie 11 were male) rather than reporting percentages, which may suggest to readers that these findings are more representative/generalizable to a wider population, which we are not sure they are. | All percentages in the results section have been changes to Ns. | Pg 5, lines 100-106 |
| Are you able to provide information regarding how many participants experienced dose reductions vs those who experienced discontinuation? Are you able to provide any info regarding average size of the dose reduction or discontinuation? | This is a great point and we wish we could include more detailed information here. Unfortunately, determining dose reduction versus discontinuation and the average size of reduction/discontinuation is part of the analysis that we plan to run as part of the larger COPING analysis. As such, we have not completed that analysis yet so are unable to include that data here. | N/A |
| Consider expanding discussion of the fact that HIV-related factors are not included in the qualitative analysis (line 95). Was it necessary to enroll HIV infected participants or do you believe that the results would have been similar with a non-HIV infected population. Please provide further justification one way or the other. | This is a great point. We edited a sentence into our results to further discuss this: "Because of this, even though the population was comprised of PLWH, our analysis did not result in HIV-specific themes." Furthermore, while we don't know if the results would have been different if the interviews had been conducted with non-HIV infected patients, it's certainly worth stating the complexity that this population may experience when assessing their HIV pain and pain management regimens. We decieded to add an explanation to our limitations section, stating: "While participants did not associate HIV with pain or pain management, they may be unaware of certain factors that influence opioid effects such as the duration of treatment with opioid pain relievers." | Pg 5, lines 110-113, and Pg 24, line 602-604 |
| Please remove marijuana from the non-pharmacologic therapies section, as cannabis and its derivatives are increasingly used in medical/pharmacologic contexts. Consider referring to the substance as cannabis, which may have fewer stigmatizing connotations relative to marijuana. | We removed marijuana from the list of nonpharmacological therapies, as the reviewer is right, cannabis is a pharmacological agent. We did not reference marijuana anywhere else in the paper. In future papers, we will use the terminology cannabis instead of marijuana. | Pg 5, line 121-122 |
| Some participants quotes (participant F, for example, in the non-pharmacologic section) seem over-represented. If possible, consider diversifying the participants whose quotes are provided in the manuscript to provide a more comprehensive overview of perspectives. | We value the reviewers comment about quote distribution, however we were very deliberate in ensuring a wide distribution of quotes, in keeping with standard guidelines and best practices for writing qualitative papers: 83% of participants are referenced at least once in the manuscript (with a range of 1-5 quotes per participant - with ppt F represented by 3 quotes). We reviewed the distribution of quotes throughout the entire paper, and feel comfortable with the distribution including the 3 quotes attributed to participant F. | N/A |
| ***Conclusions*** |  |  |
| I would encourage the authors to use the conclusion to further contextualize their work given how unique it is for them to be providing detailed perspectives from individuals with substance use/HIV. These are individuals whose voices are not commonly heard in the research literature, making this a particularly important contribution. | The conclusion now states (changes pertaining to this comment shown here in bold): "When losing access to opioid therapy for chronic non-cancer pain, **HIV-positive patients with a history of substance use** pro-actively self-manage symptoms outside of the traditional medical system. In doing so, participants describe a range of rationales, facilitators and barriers that they experienced while transitioning to non-pharmacological therapies, illicit OPRs, heroin and/or stimulants. Patients may experiment with a range of pain management modalities including nonpharmacological therapies and illicit substance use. **For this patient population, illicit drugs are a common remedy, including both opioids and stimulants.** When providers make any change to patients’ long-term opioid therapy, a holistic and patient-centered approach should be considered." | Pg 24-25, lines 608-616 |
| Seems worth revisiting here the fact that the common tropes were unrelated to HIV infection or HIV-specific care. Can you please provide some additional context to the brief discussion of this in the results section, see comment above. | We rephrased a portion of he discussion section to further emphasis that participants did not connect their HIV to their pain management experiences. We hope this will draw attention to the fact that the common tropes in our results section were not related to HIV. The sentence now states, "Although our participants did not directly link their pain or pain management to HIV, they may be more likely than other patients to have a long history of receiving opioid therapy and thus face more challenges with transitioning off of opioids (22,32)." | Page 23, line 570-575 |
| Can the authors please provide some further clarification and summarization about the respective rationales, facilitators, and barriers to the pharmacologic, non-pharmacologies and illicit substance use self-management strategies participants reported? | We now added this sentence to the conclusion to further emphasize the rationale, facilitators and barriers to using nonpharmacological therapies and illicit substances. "In doing so, participants describe a range of rationales, facilitators and barriers that they experienced while transitioning to non-pharmacological therapies, illicit OPRs, heroin and/or stimulants." | Page 25, lines 610-612 |
|  |  |  |
| **COMMENTS** | **RESPONSE** | **LOCATION OF REVISION** |
| **REVIEWER 2** |  |  |
| ***Overall*** |  |  |
| Overall this was an excellent paper and interesting to review. I learned a lot about the patient experience following opioid taper or cessiation that will impact my clinical practice. I do have some suggestions prior to publication for the authors. One thing that struck me was that there was no mention of fentanyl in the participant quotes. In other parts of North America the risk of fatal overdose is so great as the heroin supply has been replaced by fentanyl. This makes the results of your study even more compelling. I am not sure how to fit this in but it is an observation I wanted to share. | N/A | N/A |
| Is the period supposed to be before or after the reference parenthesis? I would check with the journal, usually I have seen the period before. | Thank you for catching this - according to Vancouver style the period is supposed to come after the citation. We have made this change throughout the document. |  |
| ***Abstract*** |  |  |
| In the background or methods please state the aim of the study. | We changed the final sentence of the background section of the abstract and the final sentence of our introduction to state our study aim: "The aim of this study is to qualitatively explore chronic pain management experiences among PLWH with a history of illicit substance use after long-term opioid therapy reductions or discontinuations to better understand patients’ rationales for and descriptions of barriers and facilitators to pain management modalities." | Abstract, page/line NA. Manuscript, pg 3, line 60-63 |
| ***Background*** |  |  |
| Line 21-24: I think justified is a strong word. Additionally, there is also epidemiological evidence that showed as the rate of opioid prescriptions increase so too did the rate of opioid misuse. This also supported this public health strategy | We changed the word "justified" to "based on" to decrease strength of word. | Pg 2, Line 22 |
| Line 24: suggest rephrasing “However, reduction in prescribing also carry risks:” to “However, decreasing the opioid dose or prescription cessation also carries risks:” | We've changes the phrase “However, reduction in prescribing also carry risks:” to “However, decreasing the opioid dose or prescription cessation also carries risks:” | Pg 2, Line 24-25 |
| Line 27: why is it paradoxical? | Changed the word "paradoxical" to "furthermore" | Pg 2, line 30 |
| Line 43-47: The first sentence is slightly repetitive from the sentence in line 24 and could be removed or included above in 24. It is reasonable to end paragraph starting on line 30, with the second sentence of this small paragraph. | We agree and replaced the end of the sentence previously in line 24 with the sentence 46-47 to reduce redundancy. | Pg 2, line 24-27 and line 46-47 |
| Has there been any other qualitative research in this area? If so how does your work build or add to this? If not, it is reasonable to say this, and would strongly support the need for this work. To accommodate these extra sentences you could consider paring down the second paragraph. | As far as the authors know, there is no research to date that qualitative explores the affects of opioid reduction within a population with HIV and a history of substance use. We've added a sentence to reflect this. "To date, there is a dearth of qualitative research exploring how long-term opioid reductions affect this vulnerable population of PLWH with a history of substance use." | Pg 3, lines 58-59 |
| ***Methods*** |  |  |
| Line 62: Could you say what the COPING study was about or at least what the acronym stands for and a reference to the original publication or study protocol? | We've included the acronym, and made the description of the study more explicit. We cannot provide a citation as the main COPING paper has not yet been published. | Pg 3, lines 67-68 |
| Line 71: Please describe how the interview guides were developed. Was it a flexible guide etc? | We included the phrase "semi-structured" to describe the interviews and reference the type of interview guide. | Pg 4, line 79 |
| How was language addressed? Were non-English speakers included? | Non-English speakers were not included. We've clarified this in our description of the inclusion criteria | Pg 4, line 71 |
| ***Results*** |  |  |
| The table and quantitative summary were clear and helpful. Overall the results were presented very clearly and the quotes chosen highlight the themes very well. | N/A | N/A |
| Line 281: Suggest starting the sentence at Participants. | We deleted the word "other" so that the sentence starts with "participants" | Pg 13, line 305 |
| Line 288-90: This quote is a bit confusing and does not strongly support the above theme. It almost sounds like he trusts the illicit market more? I re-read it several times. Perhaps it would be clearer if more of the quote was included or the interviewer question? Alternatively, perhaps there is another quote that could be used. | The reviewer is absolutely right- the participant IS saying he trusts the illicit market more. We felt this was an important point to make, as we felt people often group illicit OPRs and heroin in the same category given they are both street-based drugs. But for this participant, he actually trusted the heroin supply more than the pills. (I did not include this editorialization because I thought it was too significant a departure from the data, but I wonder if that is because in SF we have black tar heroin which is harder to mix with fentanyl, while the illicit pills are easier to mix so may have more impurity). We added a sentence to try and help clarify for readers that we are, in fact, illustrating that the participant had greater trust in the heroin supply than the illicit opioid. "For Participant K, a lack of trust in illicit OPRs was a motivating factor to transitioning to heroin." If the reviewer would still like us to sub out this quote for a different quote, we would be happy to do so. But we first wanted to improve our explanation of this quote to see if that would suffice as we feel it is an important concept to include as to why some individuals may transition from illicit ORPs to heroin. | Pg 12, line 298-299 |
| Line 329-336: Does the rest of the quote describe her overdose experience and how she survived? If so would be interesting to include this if it does. | We agree with the reviewer, it would be great to include more information here. Unfortunately, the participant's explanation of what happened after she blacked out is very vague. The interview tried to ask for more information, but the participant didn't remember what happened so was unable to provide detailed that we felt would enhance our analysis. We reviewed the transcript again but ultimately decided not to include the remainder of the quote because we felt, unfortunately, that it was not additive. | N/A |
| Line 402-407: This quote does not clearly capture the concept of harm reduction techniques of tester shots or cautious dosing. I suggest removing this idea or including more of this quote or another quote that illustrated this concept more clearly. | We changed the introductory sentence prior to the quote to reflect the harm reduction approach the quote described, which was sourcing drug supply from the same supplier each time. The sentence now reads: "Participant K explained employing risk reduction techniques, like using a consistent and trustworthy supplier, to ensure her safety." | Pg 17 line 414 |
| Line 426: suggest replacing “household chores” with “activities of daily living”. | We've replaced the phrase “household chores” with “activities of daily living”. | Pg 18, line 440-441 |
| Line 434: I would rephrase the idea of “education” to “lack of experience with heroin” as this seems to be what the quote supports. | We've changed the phrase "lack of education" to "lack of experience" to better reflect the sentiment of the quote. | Pg 18, line 444-445 |
| Line 447-452: This part of the quote is very interesting but does not connect to the statement above. I wonder if there is a way to highlight this piece of Narcan training specifically? | We've rephrase the opening line before the quote to allow for a connection with the mention of Narcan in the quote. The phrase now reads: "Participant D had a history of injection stimulant use but had never used heroin prior to his prescription reduction and described his lack of knowledge around heroin injection, including overdose risk reduction techniques such as naloxone utilization, as a barrier..." | Pg 19, lines 455-456 |
| ***Discussion*** |  |  |
| 551-557: This seems like a new theme not previously discussed or supported by the quotes in the paper. Participants did not describe withdrawal as a driver for their using illicit opioids. I suggest this be removed or add some quotes that support this concepts. | There were two quotes in the results section that specific discussed withdrawal. We've added explanations to those quotes to make sure it's clear to the readers that managing withdrawal symptoms was, in fact, a rationale for using. The intro to the first quote (in section Rationale for OPRs) now states: "Participant N, a mixed-raced woman in her early-50s, also reported seeking an approximation for her prescription from the street to avoid withdrawal symptoms when she was unable to access timely refills..." In the OPR barriers section we also note a participant's explanation that going through withdrawal was part of what led to her overdose event. We've also reordered the final sentence in the discussion that discussed withdrawal to state, "The FDA has recognized the dangerous and potentially life-threatening consequences of uniform tapering guidelines or abrupt tapers for opioid-experienced patients and now recommends gradual, individualized tapering plans built in conjunction with patients to modulate risk of uncontrolled pain, psychological distress, withdrawal symptoms, emergence or reemergence of substance use disorder, violence, or suicide" | Pg 11, line 258 and Pg 23, lines 580-581 |
